# Supplementary figures and images for: Caspase-1 in Cx3cr1-expressing cells drives an IL-18-dependent T cell response that promotes parasite control during acute Toxoplasma gondii infection
Source: PLoS Pathog. 2024 Oct 24;20(10):e1012006. doi: 10.1371/journal.ppat.1012006 (PMC11537422; doi:10.1371/journal.ppat.1012006)

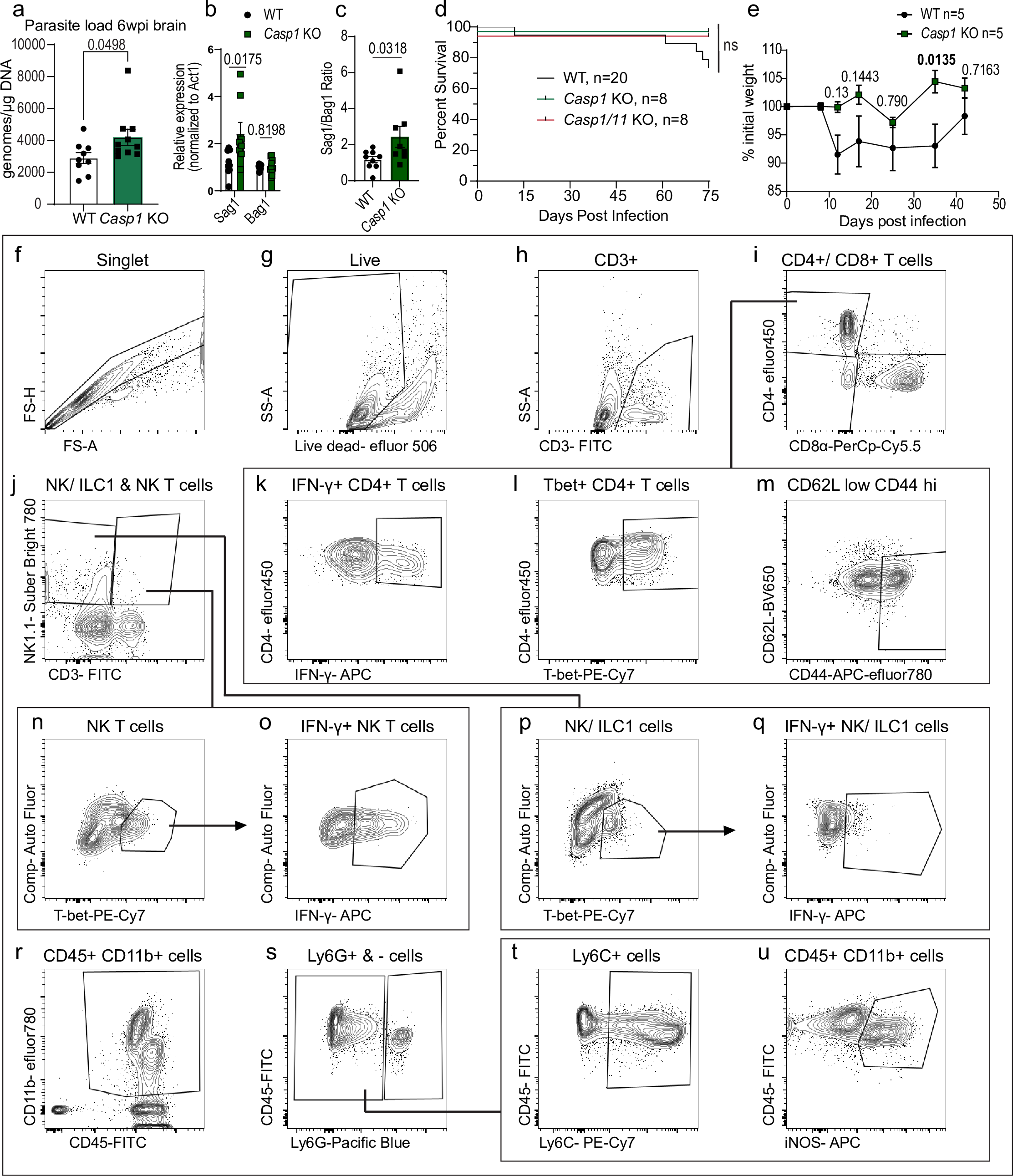

Supplement: S1 Fig — (a) qPCR analysis of T. gondii parasite load 6 weeks post-infection (6wpi) in the brain of wildtype WT C57BL/6 (n = 9) and Casp1 KO (n = 10) mice, two experiments. (b-c) RT-qPCR analysis of tachyzoite stage (Sag1) and bradyzoite stage (Bag1) specific genes in the brain at 6wpi, and non-stage specific gene (Act1) WT (n = 9), Casp1 KO (n = 8). (d) Ethical end-point curve WT (n = 20), Casp1 KO (n = 8), and Casp1/11 KO (n = 8). (e) Weights of WT (n = 5) and Casp1 KO (n = 5) mice throughout acute and chronic T. gondii infection. (f-u) Gating strategy for flow cytometry analysis of cell populations quantified in experiments. All analysis was pre-gated on singlets (f) and then on live cells (g). For T cell panels, events were gated on CD3+ (h) then on either CD4+ or CD8+ (i) these populations were then further sub-gated on IFN-γ+ (k), Tbet+ (l) and CD62Llow CD44hi (m). NK/ILC1 cells were gated on NK1.1+CD3- and NK T cells were gated on NK1.1+CD3+ (j) these two sets were then gated for negative auto-fluorescent and T-bet (n and p), the final populations were gated for IFN-γ-producing cells (o and q). Myeloid cells were gated as CD11b+CD45+ (r) then as Ly6G+ (neutrophils) or Ly6G-negative (s), the Ly6G-negative population was then gated for Ly6C and iNOS expression (t and u). Data are presented as mean ± s.e.m., p values by randomized-block ANOVA and post-hoc Tukey test (a-c), log-rank (Mantel Cox) test (d) Two-way ANOVA and Šídák’s multiple comparisons test (e). (TIF) [file ppat.1012006.s001.tif]

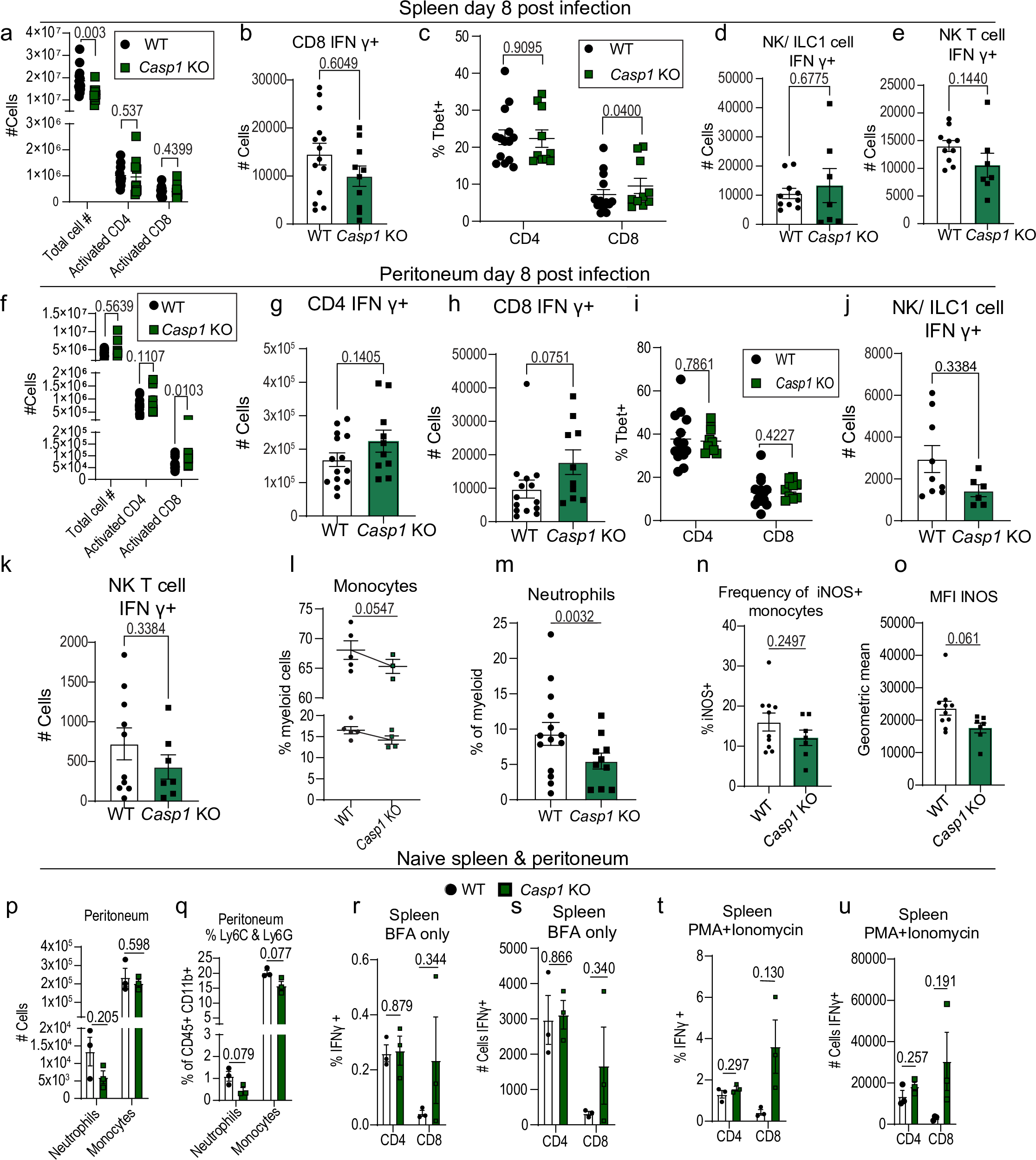

Supplement: S2 Fig — (a) Total spleen cell count at 8dpi, and number of CD62Llow CD44hi (activated) CD4+ and CD8+ T cells, three experiments, WT (n = 14) and Casp1 KO (n = 10). (b) Flow cytometry of CD3+ CD8+ IFN-γ+ T cells in spleen at 8 dpi, three experiments, WT (n = 14) and Casp1 KO (n = 10). (c) Frequency of splenic CD4+ and CD8+ T cells that are T-bet+, WT (n = 14) and Casp1 KO (n = 10). (d) Flow cytometry of CD3- NK1.1+ T-bet+ IFN-γ+ NK cells and or ILC1 cells in spleen at 8 dpi, two experiments WT (n = 10), Casp1 KO (n = 7). (e) Flow cytometry of CD3+ NK1.1+ T-bet+ IFN-γ+ NK T cells in spleen at 8 dpi, two experiments WT (n = 10), Casp1 KO (n = 7). (f) Peritoneum total cell number and number of CD62Llow CD44hi (activated) CD4+ and CD8+ T cells, three experiments, WT (n = 14), Casp1 KO (n = 10). (g-h) Flow cytometry of CD3+ (g) CD4+, and (h) CD8+ IFN-γ+ T cells in peritoneum at 8 dpi, three experiments, WT (n = 14), Casp1 KO (n = 10). (i) Flow cytometry of T-bet+ CD4+ and CD8+ T cells in the peritoneum at 8 dpi, three experiments, WT (n = 14), Casp1 KO (n = 10). (j) Flow cytometry of CD3- NK1.1+ T-bet+ IFN-γ+ NK cells and or ILC1 cells in peritoneum at 8 dpi, two experiments WT (n = 9), Casp1 KO (n = 6), one outlier removed from WT and one from Casp1 KO. (k) Flow cytometry of CD3+ NK1.1+ T-bet+ IFN-γ+ NK T cells in peritoneum at 8 dpi, two experiments WT (n = 10), Casp1 KO (n = 7). (l-n) Frequency of (l) Ly6Chi monocytes, two experiments, each experiments mean and SEM is plotted and genetic groups from each experiment are matched with a line, WT (n = 10), Casp1 KO (n = 7) (m) Ly6G+ neutrophils, three experiments, WT (n = 14), Casp1 KO (n = 10) (n) iNOS+ monocytes, two experiments WT (n = 10), Casp1 KO, in peritoneum at 8 dpi. (o) Geometric mean fluorescent intensity (MFI) of iNOS expression among iNOS+ monocytes, two experiments WT (n = 10), Casp1 KO (n = 7). (p) Number of neutrophils and monocytes in peritoneum of naïve WT (n = 3) and Casp1 KO (n = 3) mice. (q) Frequency of neut [file ppat.1012006.s002.tif]

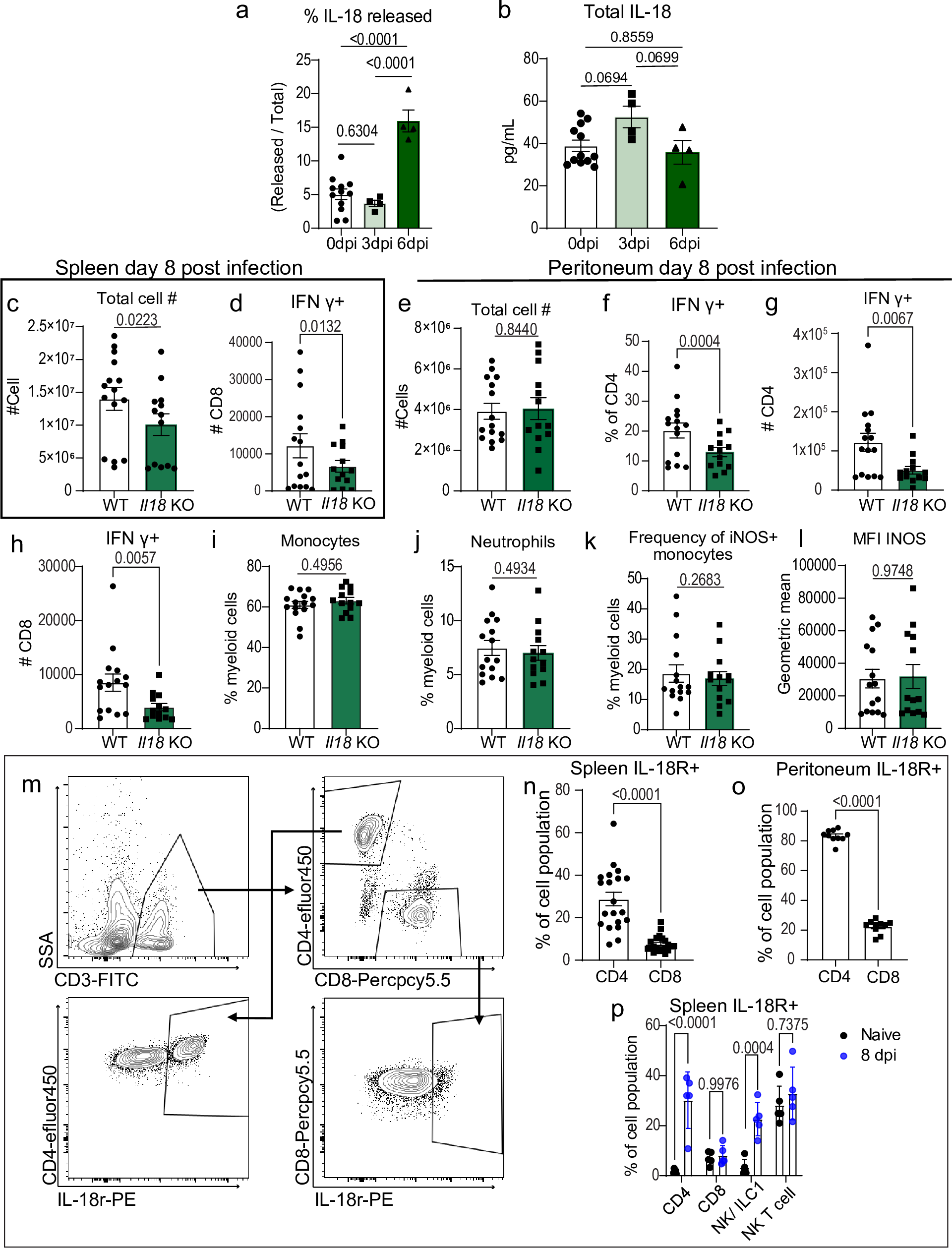

Supplement: S3 Fig — (a-b) Ex vivo cytokine release assay. (a) Percent (%) IL-18 released from equal number of PECs at 0 (n = 12), 3 (n = 4), and 6 (n = 4) dpi. (b) Total IL-18 present from equal number of PECs at 0 (n = 12), 3 (n = 4), and 6 (n = 4) dpi. (c) Number of cells in the spleen 8 dpi. (d) Flow cytometry of CD3+ CD8+ IFN-γ+ T cells in spleens 8 dpi. (e) Number of cells in the peritoneum 8 dpi. (f,g) Flow cytometry analysis of frequency (f) and number (g) of CD3+ CD4+ IFN-γ+ T cells in peritoneum 8 dpi. (h) Flow cytometry number of CD3+ CD8+ IFN-γ+ T cells in peritoneum 8 dpi. (i-k) Frequency of (i) Ly6Chi monocytes, (j) Ly6G+ neutrophils, and (k) iNOS+ monocytes in peritoneum at 8 dpi. (l) MFI of iNOS expression among iNOS+ monocytes. (c-l) three experiments WT (n = 15) and Il18 KO (n = 13). (m) Flow-gating strategy for IL-18 receptor (IL-18R) expression in CD4+ and CD8+ T cells. (n) Flow cytometry analysis of splenic CD3+ CD4+ and CD8+ T cells IL-18R expression, two experiments, (n = 20). (o) Flow cytometry analysis of peritoneal CD3+ CD4+ and CD8+ T cell IL-18R expression, one experiment, (n = 10). (p) Flow cytometry analysis of splenic CD3+ CD4+ and CD8+ T cells, NK cells and or ILC1 cells (CD3- NK1.1+ Tbet+) and NK T cells (CD3+ NK1.1+ Tbet+) IL-18R expression in naïve (n = 5) and infected mice (n = 5). Data are presented as mean ± s.e.m., p values by one-way ANOVA and post-hoc Tukey test (a and b), randomized-block ANOVA and post-hoc Tukey test (c-l and n) or Student’s t-test (o-p). (TIF) [file ppat.1012006.s003.tif]

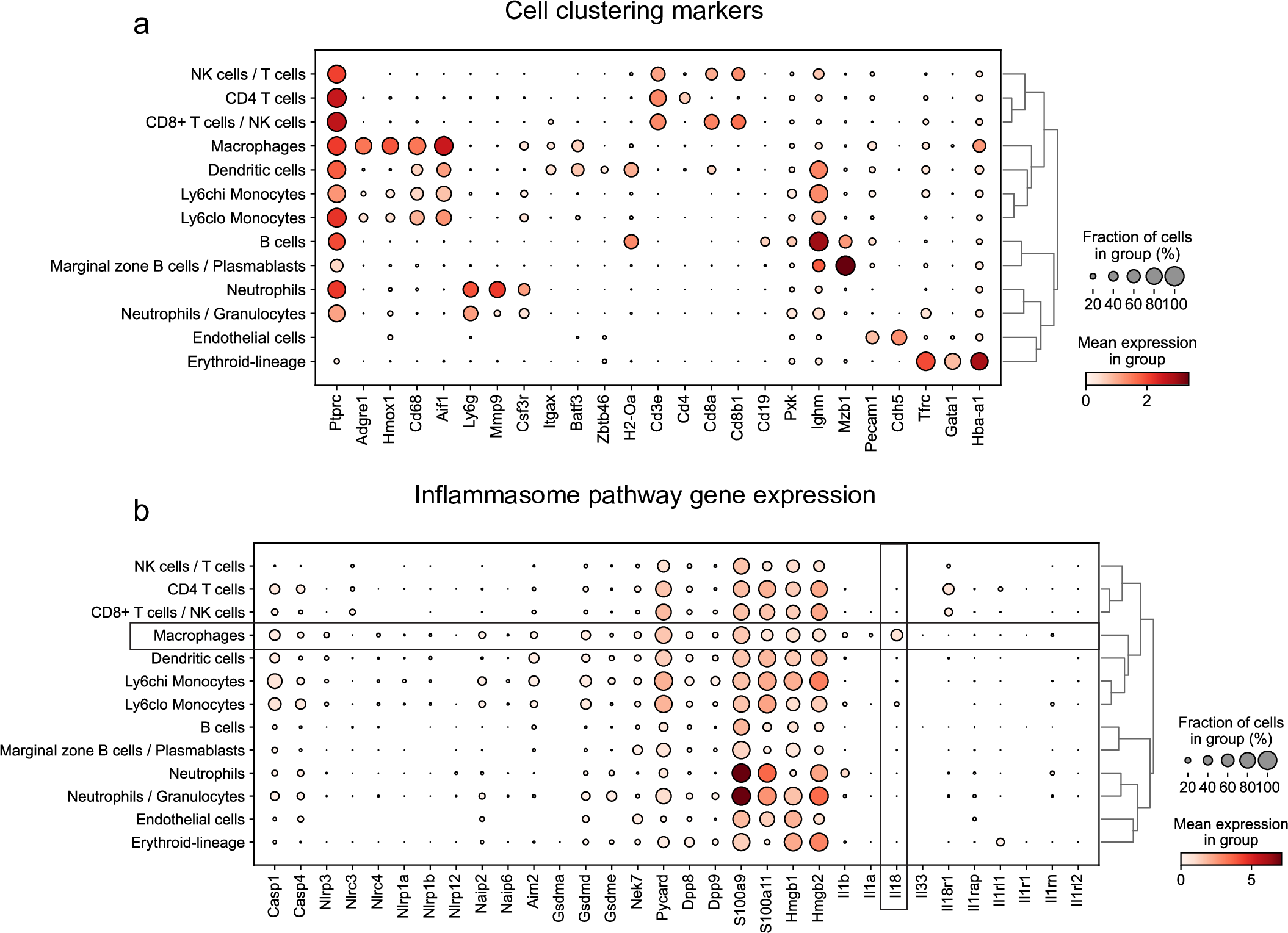

Supplement: S4 Fig — (a-b) Analysis of single cell RNA sequencing of the spleen on day 14 post-infection from [30] (a) Cell cluster segmentation markers (b) Cell type expression of manually selected inflammasome and cytokine related genes. (TIF) [file ppat.1012006.s004.tif]

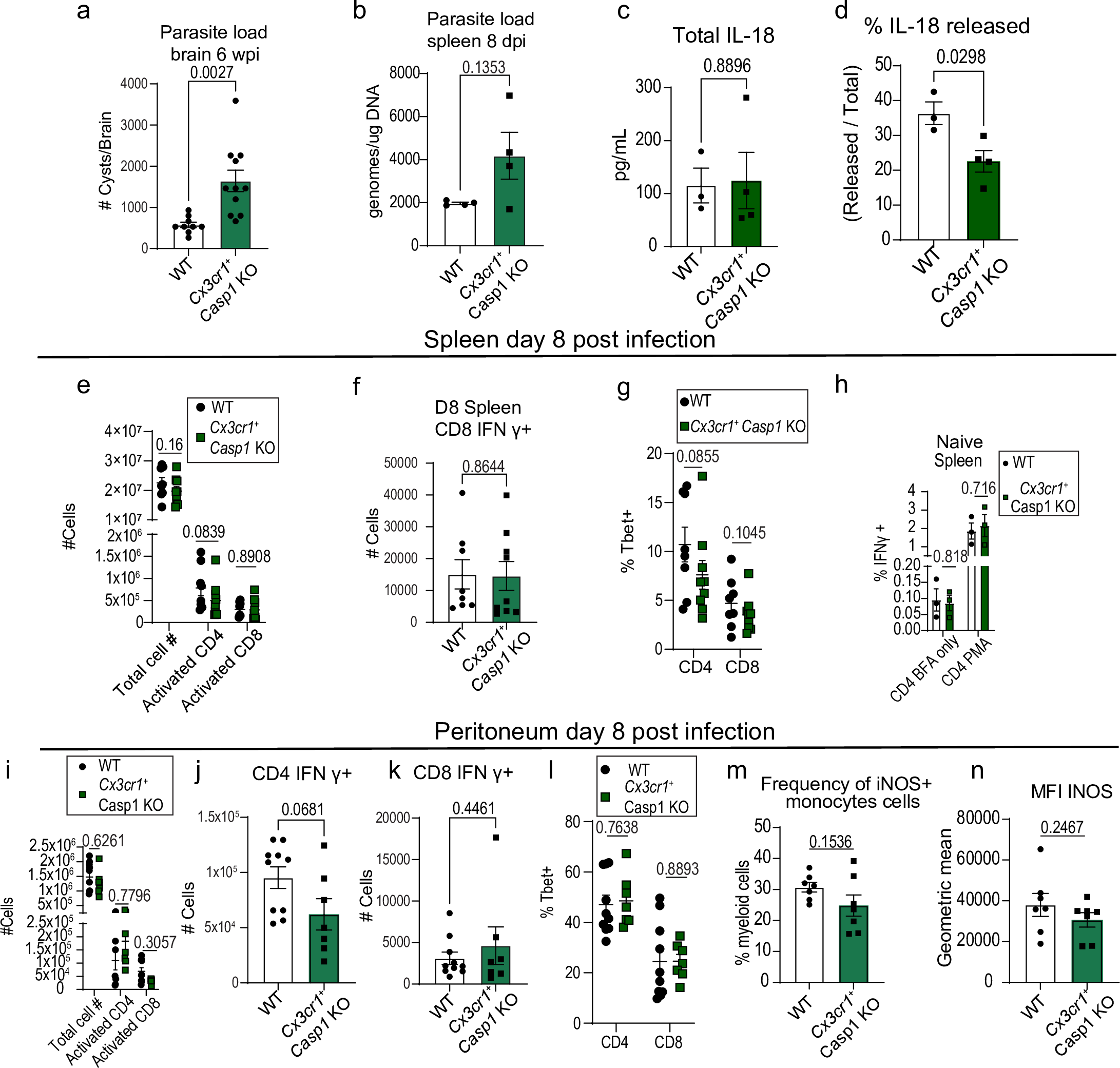

Supplement: S5 Fig — (a) Cyst counts of T. gondii parasite load 6 weeks post-infection (6wpi) in the brain of wildtype WT (n = 9) and Cx3cr1+ Casp1 KO (n = 11) mice, two experiments. (b) Parasite load in the spleen 8dpi in WT (n = 4) and Cx3cr1+ Casp1 KO (n = 4) mice. (c and d) Ex vivo cytokine release assay WT (n = 3) and Cx3cr1+ Casp1 KO (n = 4). (c) Total IL-18 present from equal number of PECs at 6 dpi. (d) Percent (%) IL-18 released from equal number of PECs at 6 dpi. (e) Splenic cell number and number of CD62Llow CD44hi (activated) CD4+ and CD8+ T cells 8dpi, two experiments, WT (n = 8) and Cx3cr1+ Casp1 KO (n = 9). (f) Flow cytometry of CD3+ CD8+ IFN-γ+ T cells in spleen at 8 dpi, two experiments, WT (n = 8) and Cx3cr1+ Casp1 KO (n = 9). (g) T-bet+ splenic CD4+ and CD8+ T cells 8dpi, two experiments, WT (n = 8) and Cx3cr1+ Casp1 KO (n = 9). (h) Frequency IFN-γ+ splenic CD4+ and CD8+ T cell in naïve WT (n = 3) and Cx3cr1+ Casp1 KO (n = 3) mice. (i) Peritoneal exudate cell number and number of CD62Llow CD44hi (activated) CD4+ and CD8+ T cells 8dpi, two experiments, WT (n = 10) and Cx3cr1+ Casp1 KO (n = 7). (j-k) Flow cytometry of CD3+ (j) CD4+ (k) CD8+ IFN-γ+ T cells in peritoneum at 8 dpi, two experiments, WT (n = 10) and Cx3cr1+ Casp1 KO (n = 7). (l) T-bet+ peritoneal CD4+ and CD8+ T cells 8dpi, two experiments, WT (n = 10) and Cx3cr1+ Casp1 KO (n = 7). (m-n) Frequency of iNOS+ monocytes in peritoneum(m) and Geometric mean intensity of iNOS among iNOS+ monocytes(n). Data are presented as mean ± s.e.m., p values by randomized-block ANOVA and post-hoc Tukey test (a, e-g, i-n) and Welch’s t-test (b, c, d and h). (TIF) [file ppat.1012006.s005.tif]

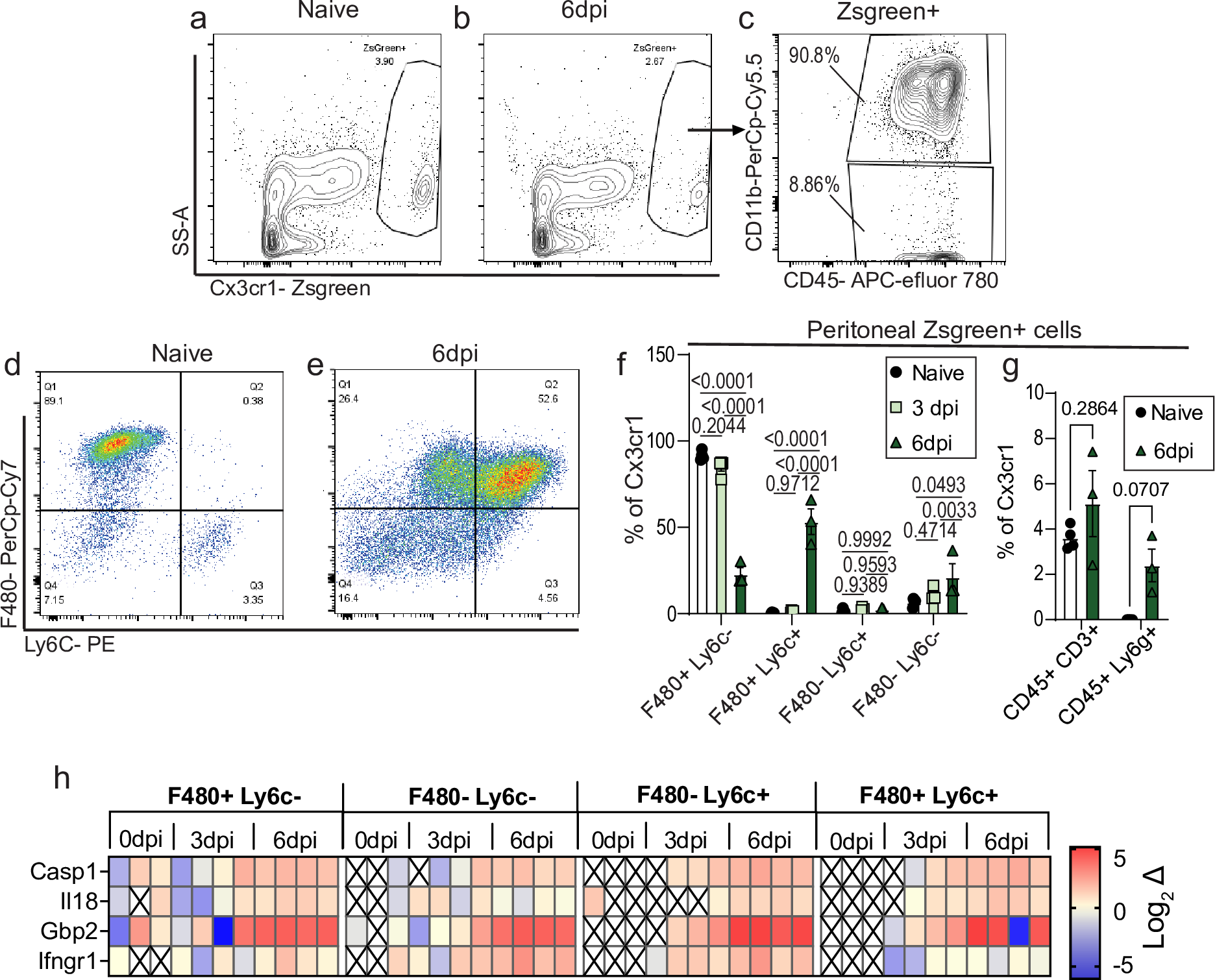

Supplement: S6 Fig — (a-h) Inducible Cx3cr1CreERT2/WT Rosa26 Ai6/Ai6 (Cx3cr1-reporter mice) were given tamoxifen chow and injected with PBS or 10 cysts Me49. (a-b) Reprehensive flow plots of Cx3cr1+ cells (ZsGreen-positive) (a) Naïve reporter mouse PECs (b) Reporter mouse PECs 6dpi. (c) ZsGreen+ cells from naïve and 6dpi were gated on CD45+ CD11b-negative or CD11b-positive. (d-e) CD45+CD11b+ cells were gated on F480 and Ly6C expression. (f) Quantification of % of ZsGreen (Cx3cr1) positive cells with/without F480 expression and with or without Ly6C expression, naïve (n = 4), 3dpi (n = 3), and 6dpi (n = 3). (g) Quantification of % of ZsGreen (Cx3cr1) positive cells CD3+ or Ly6G+, naïve (n = 4), 3dpi (n = 3), and 6dpi (n = 3). (h) qPCR heatmap for Casp1, Il18, Gbp2, Ifngr1 in Cx3cr1+ F480(+/-) and Ly6C(+/-) myeloid populations at 0, 3, and 6 dpi. Shown as log2 change relative to 0 dpi F480+ Ly6C-. Data are presented as mean ± s.e.m., p values by Welch’s t-test (f and g). (TIF) [file ppat.1012006.s006.tif]

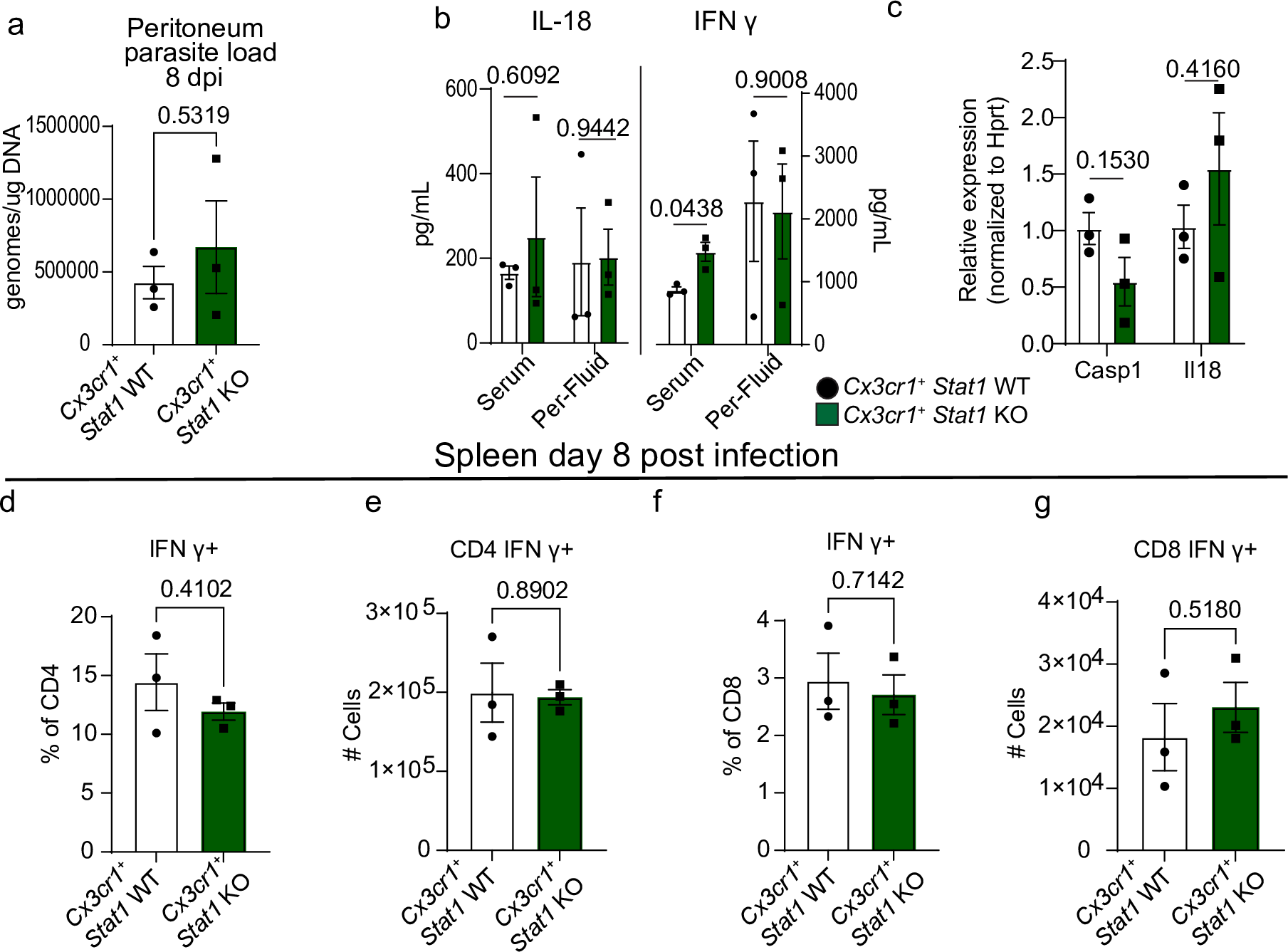

Supplement: S7 Fig — (a-g) Cx3cr1 CreERT2/WT Stat1WT/WT and Cx3cr1 CreERT2/WT Stat1fl/fl mice were administered 5 doses of tamoxifen i.p. at 20 g/kg. After two weeks, 10 cysts of Me49 was administered i.p. (a) Peritoneal parasite burden at 8 dpi. (b) IL-18 and IFN-γ cytokine levels in serum and peritoneal fluid (Per-Fluid) at 8 dpi. (c) qPCR of Casp1 and Il18 at 8 dpi in PECs. (d-g) Flow cytometry of splenocytes incubated in BFA for 5 hours. (d) Percent of CD4+ T cells making IFN-γ. (e) Number of CD4+ T cells making IFN-γ. (f) Percent of CD8+ T cells making IFN-γ. (g) Number of CD8+ T cells making IFN-γ. Data are presented as mean ± s.e.m., p values by Welch’s t-test (a-g). (TIF) [file ppat.1012006.s007.tif]
