# Supplementary material for: Caspase-1 in Cx3cr1-expressing cells drives an IL-18-dependent T cell response that promotes parasite control during acute Toxoplasma gondii infection
Source: PLoS Pathog. 2024 Oct 24;20(10):e1012006. doi: 10.1371/journal.ppat.1012006 (PMC11537422; doi:10.1371/journal.ppat.1012006)
Supplement: Attachment — Submitted filename: response to reviewer IB 1062024.pdf [file ppat.1012006.s010.pdf]

We thank the reviewers for their comments and suggestions to strengthen our manuscript titled *Caspase-1 in Cx3cr1-expressing cells drives an IL-18-dependent T cell response that promotes parasite control during acute Toxoplasma gondii infection.* We have completed experiments suggested by reviewer comments and have modified the text and figures. Specific changes and responses to reviewer comments are below in blue text.

Part I – Summary

Please use this section to discuss strengths/weaknesses of study, novelty/significance, general execution and scholarship.

Reviewer #2: The revisions of the manuscript by Babcock et al. only marginally address the main questions/concerns raised by the reviewers. Although the editors asked for Major Revisions, none of the 5 main Figures has been modified and/or improved with new information. New information provided in Supplementary Figures hardly enhances the original data and raises even more questions.

In response, we have added new data to Main Figure 2.

The nature of the Cx3cr1 population remains obscure. To make things even more complicated, the authors add on a possibility that steady-state expressing Cx3cr1 cells are responsible for IL-18 production (although in the experiment shown in Response Figure 1, the frequency of IFN-g+ CD4 T lymphocytes was not significantly reduced in spleen, in contrast to observations made in infected Casp1 KO mice). Moreover, based on data in Figure S5, Cx3cr1 expressing cells in PEC and spleen in infected mice are very different from the ones in steady-state (as expected inflammatory monocytes vs tissue resident macrophages). Again, the Casp1 expressing population could be the former or latter, both, or neither.

To address this question, we opted to sort *Cx3cr1*-expressing cells and perform qPCR for Casp1. We found that all *Cx3cr1*-expressing populations express Casp1. The data is now in Figure S6h. We further address the implications of these experimental results below under major revisions Reviewer 2 point #2.

Similarly, based on Figure S2, the defect in Casp1 KO mice could be due to the lack of splenic CD4+ Th1 lymphocytes, or to an increase in frequency of CD8+ T cells that express Tbet (observed both in spleen and PEC). We just do not know.

Differences in responses between PEC and spleen specifically in Casp1 KO or Cx3cr1+ Casp1 KO (but not IL-18 KO) infected with T. gondii remain poorly explained. Perhaps instead of looking only at one time point, the authors should have performed a kinetics

study with earlier time points (e.g. day 3, 5 and 8) and examined all possible sources of IFN-g (e.g. ILC, gd T, NK, cells).

We have added text to the discussion to try to better explain differences observed in the spleen versus peritoneum in lines 379-382. Additionally, we have performed a time course experiment utilizing the 0dpi, 3dpi, and 6dpi timepoints to determine when IL-18 is released (Fig2a, S3a-b) and expressed (S6h) and found that IL-18 is not released by PECs or increased in serum and peritoneal fluid until 6 dpi. Although IL-18 RNA and protein is present in cells at 0dpi, 3 dpi and 6 dpi. This data suggests that 8 dpi is reasonable timepoint to determine the effects of IL-18 on host immune response during acute *T. gondii* infection.

I agree with the authors that there is too much variation between individual data points, which may potentiate minor and/or cancel out real differences between tested groups when results from several experiments are pooled.

Reviewer #3: The authors have responded appropriately to the previous comments. I do not have any further comments.

Thank you!

Reviewer #4: This is a very thorough revised manuscript that provides important information regarding the role of CX3CR1-positive macrophages in regulating caspase-1 dependent IL-18 response during T. gondii infection. Not only did the authors develop precise mouse models to clarify a very confusing field, but they also defined the role of caspase-1 during T. gondii infection. These are undoubtedly important and impactful results. In addition, while this reviewer did not evaluate the initial submission, I feel that the authors fully addressed the previously identified deficiencies.

Thank you very much!

Part II – Major Issues: Key Experiments Required for Acceptance
Please use this section to detail the key new experiments or modifications of existing experiments that should be <u>absolutely</u> required to validate study conclusions.

Generally, there should be no more than 3 such required experiments or major modifications for a "Major Revision" recommendation. If more than 3 experiments are necessary to validate the study conclusions, then you are encouraged to recommend "Reject".

Reviewer #2: The authors did not satisfactory addressed two major issues:

For the record, there was no concern “1”.

2. Characterize the population of Caspase-1 expressing Cx3cr1+ cells.

To address which of the *Cx3cr1*-expressing populations express caspase-1 we measured mRNA expression of Casp1. We have not found a reliable flow cytometry antibody for caspase-1 or had success with fluorometric activity assays for caspase-1 activity such as FLICA. To address *Casp1* expression, we sorted Cx3cr1$^+$ cells from the peritoneum at baseline (0 dpi), 3 dpi, and 6 dpi into the four predominate myeloid populations that Cx3cr1+ cells comprise (F480$^+$Ly6C$^-$, F480$^+$Ly6C$^+$, F480$^-$Ly6C$^+$, and F480$^-$Ly6C$^-$). We then performed RT-qPCR to characterize which of the Cx3cr1$^+$ cell populations express Casp1 throughout a time course of acute infection. We found that all four populations express Casp1 and Il18 making them all putative cell populations relevant to the current study. This characterization yielded temporal and cell type specific information on Cx3cr1$^+$ cells and their Casp1 and IL-18 expression. This data can be found in Figure S6h.

3. Demonstrate that IL-18 expression is upstream (of IFN-g) and not down-stream from IFNgR-gamma signaling.

To address this question, we needed a way to interfere with IFN-gamma signaling that would not affect parasite control. IFN-γ is the critical cytokine that promotes protection and parasite control during acute *T. gondii* infection. Therefore, manipulation of this pathway with blocking antibodies increases parasite burden and can lead to uninterpretable results. Therefore, we had to use Cx3Cr1$^{CreERT2/WT}$Stat1$^{fl/fl}$ mice. By, using the mice we circumvented the issues of global blockade of IFN-γ by specifically targeting *Stat1* in Cx3cr1-expressing cells. Using the Cx3Cr1$^{CreERT2/WT}$Stat1$^{fl/fl}$ mice we did not observe a dependence on IFN-γ signaling to impact Il18 expression (S7c), Casp1 expression (S7c), IL-18 serum and peritoneal fluid levels (S7b), IFN-γ serum or peritoneal fluid levels (S7b), CD4 or CD8 splenic T cell production of IFN-γ (S7d-g), or acute control of parasite burden (S7a). Similar results were previously published by our group (Cowan et al 2022), showing that STAT-1 signaling in tissue macrophages does not impact the early control of infection or the acute T cell response. Interestingly, although not significant, *Casp1* expression trended towards being lower in mice with Stat1 deficient Cxc3cr1-expressing cells (S7c). This along with Casp1 expression increasing along with IFN-γ-induced gene Gbp2 (S6h) suggests a potential role for IFN-γ to regulate the expression of *Casp1*. Nevertheless, we found this upregulation to be dispensable for IL-18 release and its ensuing impact on the immune response. Although, the Cx3Cr1$^{CreERT2/WT}$Stat1$^{fl/fl}$ mice mouse was the optimal model to address the raised question, it does have the caveat that IFNγR signaling on resident macrophages impacts their disappearance from the peritoneal cavity and myeloid cell dynamics in the peritoneum at 5 dpi (Martin et al 2024).

An additional experiment we carried out to address the concern was to isolate PECs from *Cx3cr1+ Casp1* WT and KO mice at 6 dpi and assay the total IL-18 present to determine whether the decrease in IFN-γ seen in these mice impacts the cellular abundance of IL-

18. We observed no difference in total IL-18 present between *Cx3cr1+ Casp1* WT and KO PECs at 6 dpi but did observe significantly less IL-18 being released from *Cx3cr1+ Casp1* KO PECs compared to their controls (S5c-d). This data would suggest that the systemic decrease in IL-18 is due to *Cx3cr1+ Casp1* KO cells inability to release IL-18 and is not due to IFN-γ regulation of IL-18. We agree that there are many interesting questions regarding the regulation and induction of alarmin release during acute infection, but our data does not support a role for interferon signaling upstream of IL-18 release in this infection.

Reviewer #3: (No Response)

Reviewer #4: (No Response)

Part III – Minor Issues: Editorial and Data Presentation Modifications
Please use this section for editorial suggestions as well as relatively minor modifications of existing data that would enhance clarity.

Reviewer #2: If n=1, it cannot be a representative graph.
Figure S5 (j) Representative graph of splenic ZsGreen (Cx3cr1) positive cells from naïve (n=1) and 8dpi (n=2) showing their distribution within varying F480 and Ly6C expressing populations.

This has been removed.

Panel K in Figure S5 might be out of place (?). This panel, in agreement with data depicted in the Figure 1 panel h, shows a lower frequency of inflammatory monocytes, the essential effectors population for control of T. gondii replication. However, this was not observed in infected Cx3cr1 Cas1 KO mice (figure 4h), again showing that correlations between different mouse strains and sets of pre-selected immunological parameters cannot be used as evidence for causation.

Figure S5 has been removed.

The conclusion that Casp1 KO mice do not lose weight during the infection is based on 1 time point (day 35 post infection).

We have specified in the Results section at which timepoint *Casp1* KO mice showed significantly different weight loss compared to WT mice. This can be found on line 134.

Using scientific numbers for the graphs that depicts cell numbers in millions should be applied in all such panels.

Graphs have been changed.

Reviewer #3: (No Response)

Reviewer #4: I have only two minor recommendations for the authors:

1.Line 86: Please add ILC1 as IFN-γ producers downstream of IL-12 and provide the appropriate references for T. gondii infections.

We have done this, thank you!

2.One puzzle (no need to address experimentally) is the relatively large amount of IL-18 in CX3CR1xCasp1fl/fl mice (Fig. 4D). This suggests that while CX3CR1-positive macrophages are contributing to IL-18 production, other cells can produce this cytokine as well. There is no need to perform any additional experiments; just acknowledge this fact and compare the data to the previously published Casp1x11 complete KO mice.

This is an interesting observation; we have commented on it in the discussion on line 330 and on line 395.
