# Supplementary material for: Caspase-1 in Cx3cr1-expressing cells drives an IL-18-dependent T cell response that promotes parasite control during acute Toxoplasma gondii infection
Source: PLoS Pathog. 2024 Oct 24;20(10):e1012006. doi: 10.1371/journal.ppat.1012006 (PMC11537422; doi:10.1371/journal.ppat.1012006)
Supplement: Attachment — Submitted filename: response to reviewer comments 6122024 v2.pdf [file ppat.1012006.s009.pdf]

We thank the reviewers for their comments and suggestions to strengthen our manuscript titled *Caspase-1 in Cx3cr1-expressing cells drives an IL-18-dependent T cell response that promotes parasite control during acute Toxoplasma gondii infection.* We have completed experiments suggested by reviewer comments and have modified and corrected text and figures. Specific changes and responses to reviewer comments are below in blue text.

Reviewer #1: s is an interesting study where the authors report that the critical function of Caspase 1 in CX3CR1 expressing macrophages is to drive IL18 release to maintain CD4+ T cell IFN-γ production and play a protective role during acute T. gondii infection. Previously the same group reported that IL-1α release from microglia (CX3CR1 positive cells) promotes myeloid cells recruitment into the infected brain and controls parasites during chronic T. gondii infection (Batista et al, 2020, Nat Communications). However, in the current study they did not observe myeloid cell recruitment. Overall, the manuscript is well written, and the conclusions are supported by the data. There are some concerns that need to be addressed by the authors as mentioned below.

Reviewer #2: The study performed by Babcock et al. focuses on examining how a deficiency in Caspase-1 may affect the immunity in Toxoplasma gondii infected mice. The authors observed only an increase in the parasite burden that was, nevertheless, well tolerated since survival was unimpaired and infection progressed to the chronic phase. The authors did not test if a higher dose of the parasite and/or oral route of infection will have a more significant impact on host protective immune responses in infected Caspase-1 deficient animals.

The data presented have several gaps.

- While the parasite burden is higher in the peritoneum (site of infection), the authors focused on a small change in IFN-gamma producing CD4+ T lymphocyte in the spleen.

This is a completely accurate observation. We only observed the T cell defect in the spleen. We did observe a lot of variation in the $CD4^+$ T cells in the peritoneal cavity at this timepoint. One hypothesis is that the pertinent IL-18 releasing cell may not be present in the peritoneum and may only act locally in the spleen. We have included a sentence in the discussion addressing this point and provide a potential hypothesis as to why we specifically see an IFN-γ defect specifically in the spleen is on **line 360-363**.

- It appears that IFN-g+ CD4+ were analyzed without any restimulation, but also without prior in vivo Brefeldin A injection, which might not be the most accurate way to examine their effector function.

We apologize for the lack of clarity. These studies utilized ex vivo brefeldin A treatment prior to flow cytometric analysis. While it may not capture as much IFN-gamma production as in vivo injection of brefeldin A, we believe it provides an assessment of active cytokine production in comparison to restimulation. Furthermore, with brefeldin A

alone we were able to reproducibly detect T cell populations producing IFN-γ, providing confidence that brefeldin A alone ex vivo was sufficient for our studies.

- According to Material and Methods, the authors used anti-Tbet Ab in their panel, as well as CD44 and CD62L. It would be important to show whether there is a difference in the number of activated CD44+ CD4+ T cells. What are the frequencies of T-bet+ populations?

We have provided this data in the manuscript, in **(supplemental figure 2a,c,f,i,) and (supplemental figure 4e,g,I,l).** In *Casp1* KO mice we see a slight increase in the number of T-bet+ CD8 T cells in the spleen, we also see more activated CD8 T cells in the peritoneum of *Casp1* KO mice. We have noted these changes on **lines 148 and 152**.

Similarly, while the frequency of IFN-g+ CD4+ T cells appears to be only slightly decreased, their absolute number is much more affected. Is spleen smaller in Caspase-1 KO mice when compared to ones from WT group on day 8 post-infection? What was the parasite burden in spleens from WT and KO animals?

Caspase-1 KO and *Il18* KO mice had fewer cells in their spleen which could lead to the increased difference in absolute number of CD4 T cells making IFN-γ. We have included this data in **supplemental figure 2a, supplemental figure 3a, Figure 3c, supplemental figure 4e**. Additionally, we have included parasite burden in the spleen for *Cx3cr1*$^{+}$ *Casp1*KO mice compared to WT mice in **supplemental figure 4d.**

- Next, authors show that in the peritoneum there is a decreased number of monocytes and neutrophils. Again, although they employed anti-iNOS Ab in their panel (line 457), we don't know if the production of iNOS was diminished, which could contribute to increased parasite load in Caspase-1 deficient group. What was the frequency of the recruited inflammatory macrophages in peritoneum? A more comprehensive phenotyping of cells in peritoneum as indicated above for splenocytes needs to be provided.

Thank you for the suggestion, we analyzed the iNOS production of myeloid cell in the peritoneum and did not see a significant difference in frequency of myeloid cells producing iNOS or in the iNOS mean expression levels by qPCR, data can be found in **(supplemental figure 2n-o, 3i-j, and 4m-n)**. We did not notice consistent IFN-γ defects in the peritoneum it was not unexpected that iNOS levels were not impacted. Additionally, we have included peritoneum monocyte and neutrophil frequencies in **(supplemental figure 2l-m and 3g-h).**

Interestingly, the ratio between number of monocytes vs. neutrophils looks quite comparable between the WT and Caspase-1 KO animals (~25:1).
Sometimes minor changes that can reach statistical significance may not have

biological significance. A similar drop in the frequency of neutrophils was observed in infected IL-1RAP KO as in Caspase-1 KO mice but with no impact on the parasite load. Moreover, the "lower" number of neutrophils in infected IL-1RAP KO mice (Fig. 3) is the same as in infected control WT group in Figure 1.

There is a large amount of variability in the immunologic response obtained from independent infections. Because of this, comparing data across independent experiments is not a reasonable comparison. For this reason, we use a block ANOVA which enables group comparisons between independent experiments by accounting for the variance between dates of infection. For clearer presentation of the results, we have included the percentages of monocytes and neutrophils recruited to the peritoneum in **(supplemental figure 2l-m and 3g-h)** and p values throughout the manuscript**.**

>In the rest of the manuscript, authors explore the role of IL-18 during T. gondii infection. They show that the mice lacking IL-8, as in Caspase-1 KO group, have decreased (-20%) serum IFN-gamma levels, which correlates with a smaller pool of IFN-gamma+ CD4+ T cells in spleen. The same observation (Fig. 2 vs 4) was made in mice that lack expression of Caspase-1 in Cx3cr1+ cells targeted as a potential source of IL-18. However, in the latter group, substantial systemic levels of IL-18 remain detectable (in contrast to IL-18 KO animals; much higher than in Caspase-1 KO and not very different from the level observed in WT control group in Fig. 1d).

We agree that we were expecting a larger decrease in IL-18 in the *Cx3cr1*$^+$ and *Casp1* KO studies. Regardless, this ~50% reduction in IL-18 serum level was associated with reduced serum IFN-γ similar to the 100% absence of IL-18 in the IL18KOs. *Clark et al., 2023. Cell press* describes that IL-18 cytokine responses are tightly regulated by IL-18 binding protein suggesting only a slight change in IL-18 levels is necessary to see its maximum effect. We have included this point in the discussion on **line 373-376.** Additionally, similar levels of decreased IL-18 serum levels have been observed by (*Gorfu et al., 2014. mBio*) in NLRP1 and NLRP3 KO mice infected with *T. gondii*.

>Moreover, exogenous IL-18 administration augmented neither serum IL-18 nor IFN-gamma levels in mice that lack expression of Caspase-1 in Cx3cr1+ cells, but it did increase the frequency of IFN-gamma+ CD4+ T cells in spleen that correlates with decreased parasite burden in peritoneum.

The reviewer makes an important point. Although IL-18 administration did not make serum levels of IL-18 or gamma significantly different from the KO group it did return the levels of IL-18 and IFN-γ to be no different from WT levels, suggesting we had a partial rescue of IL-18 and IFN-gamma. We have revised the text on **line 281** to make it clear that we observed only a partial rescue.

>Based on the well-documented IFN-gamma-stimulatory role of IL-18, it is expected that

rIL-18 would improve the Th1 immunity in mice that lack expression of Caspase-1 in Cx3cr1+ cells; very likely rIL-12 treatment would do the same. It is not clear why the authors didn't try to rescue IL-18 deficient animals with rIL-18 treatment.

We did not think this was a necessary experiment as we did the IL-18 rescue in the *Cx3cr1*$^{+}$ caspase-1 KO mice which specifically lack complete IL-18 levels. We do not think the additional knowledge gained from the proposed experiment would add any new information to the current study.

>In addition, because IFN-gamma regulates the transition of inflammatory monocytes to Cx3cr1+ macrophages, it is important to show the frequency of the latter population throughout the study. Otherwise, one can argue that, due to some other yet-to-be defined defect, the infected Crisper-1 KO mice are making less IFN-gamma that impairs development of Cx3cr1+ macrophages, which leads to lower serum levels of IL-18.

The reviewer's point is well taken. We have included an analysis of *Cx3cr1* expressing cells throughout the course of acute infection in new **supplemental figure 5**. To address your alternative hypothesis, we have generated data using an inducible Cx3cr1-creERT2 x Caspase-1 fl/fl mice treated with tamoxifen 4 weeks prior to infection to specifically knockout Casp1 in the Cx3cr1 expressing cells that exist prior to infection and at the time of tamoxifen treatment. In this preliminary experiment, we observe the same defect as we do with the constitutive Cx3cr1-Cre mouse line (**Response figure 1**). These data would suggest that the pertinent *Cx3cr1* population is long-lived and developed prior to infection-induced IFN-γ.

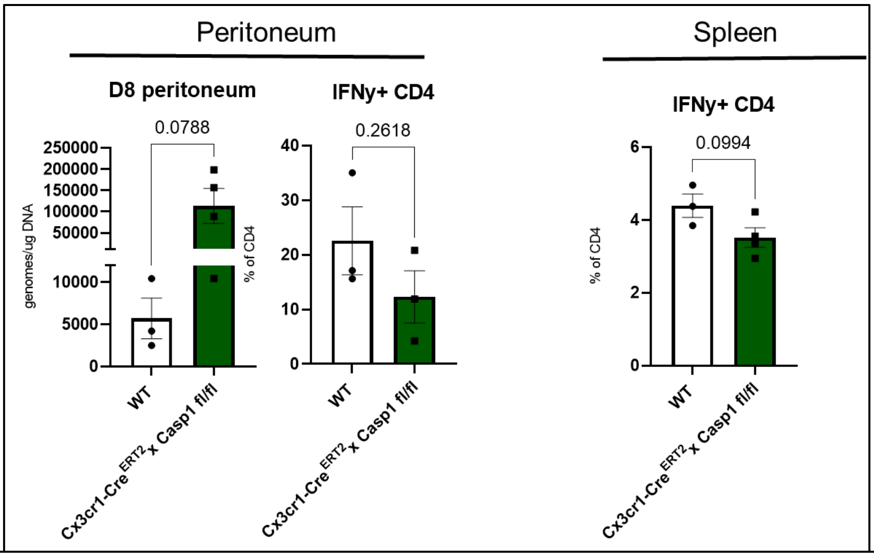

Response figure 1. Inducible Cx3cr1$^{CreERT2/WT}$ x Casp1 $^{fl/fl}$ and Cx3cr1$^{WT/WT}$ x Casp1 $^{fl/fl}$ mice were treated with 5 shots of tamoxifen solubilized in corn oil at a concentration of 20 mg/mL to excise *Casp1* from preexisting Cx3cr1-expressing populations. Four weeks post tamoxifen treatment mice were infected with 10 cysts Me49 parasite. Eight days post-infection spleens and peritoneum were harvested. To measure IFN-γ production, single cell suspensions were incubated with brefeldin A *ex vivo* for 5 hours. Data is from one experiment, each group has an n=3, statistics are Welch's t-test.

>In Figures 4 and 5, mice that lack expression of Caspase-1 in Cx3cr1+ population are referred to as Mf Casp KO (for simplicity?). Is it or is it not Mf Casp KO? After reading the manuscript, the tittle “Caspase-1 producing Cx3cr1+ cells…” sounds ambiguous. The authors should stick to one or the other interpretation.

We agree with the reviewer and have changed the nomenclature throughout the text from Macrophage *Casp1* KO to *Cx3cr1*+ *Casp1* KO.

>Once more, better quantification of lymphoid and myeloid populations in spleen and peritoneum from mice that lack Caspase-1 expression in Cx3cr1+ cells would strengthen this manuscript. For example: How many Cx3cr1+ myeloid cells are in spleen vs. peritoneum and in KOs vs. WT mice?

Thank you for the recommendation we have addressed these points in your comments above.

Reviewer #3: Here, Babcock, Harris and colleagues investigate the role of caspase-1 in the immune response to live T. gondii infection. Using both whole body and cell-type restricted deficiency models, they conclude that caspase-1 is required for optimal immune responses and the effect of caspase-1 is principally mediated by IL-18 produced and released from a CX3CR1-expressing cell.

>The chief consequence observed downstream of caspase-1 deficiency seems relatively subtle/minor in nature, ie., subtle increases in parasite loads in the acute and chronic phases of infection. It is not clear whether the increase is parasite loads is mainly an increase in bradyzoite loads or also accompanied by increased tachyzoite numbers in the CNS. Given that no changes were observed in overall mortality, it is more likely that the mice were able to achieve sufficient control of chronic infection, without a clear increase in reactivation of lytic phase infection.

Thank you for the comments, we have included data from the chronic phase of infection (6 weeks post infection) showing the relative ratio of bradyzoite (Bag1) to tachyzoite (Sag1) gene expression in the infected brains of WT and *Casp1* KO mice in **supplemental figure 1b-c.** In *Casp1* KO brains we observed a significantly higher ratio of tachyzoite gene expression relative to bradyzoite gene expression. These data suggest an incomplete ability to control *T. gondii* in the brain although we agree that sense *Casp1* KO mice do not die the lack of parasite control observed is not sufficient or extreme enough to induce death.

Part II – Major Issues: Key Experiments Required for Acceptance
Please use this section to detail the key new experiments or modifications of existing experiments that should be absolutely required to validate study conclusions.

Generally, there should be no more than 3 such required experiments or major modifications for a "Major Revision" recommendation. If more than 3 experiments are necessary to validate the study conclusions, then you are encouraged to recommend "Reject".

Reviewer #1:

1. To confirm that in naïve mice lacking Caspase 1, IL18, cx3cr1 expressing macrophages had no effect on the presence of CD4+ T cell IFN-γ in the spleen it is strongly recommended to add flow cytometry results before infection (Day 0) in Fig 1e, 2d and 4e.

Thank you for the suggestion, we agree that this will make the manuscript stronger. We have included naïve studies for *Casp1* KO and *Cx3cr1*-cre *Casp1* KO and the relevant control mice looking at T cell IFN-γ in spleen under conditions of Brefeldin alone as well as Brefeldin plus PMA and ionomycin in **supplemental figure 2r-u.** We have also included naïve experiments for monocyte and neutrophil number and frequency at base line in the peritoneum in **supplemental figure 2p-q** as we think this is also an important naïve measure to have.

2. To confirm that Casp1 in CX3CR1 expressing macrophages play a major role in the regulation of CD4+ T cell IFN-γ the authors should perform an adoptive transfer of CX3CR1 expressing macrophages into Casp1 KO mice to check if it restores CD4+ T cell IFN-γ production and control parasite burden.

This would be a powerful experiment, but we have concerns about the feasibility of the proposed experiment. With further analysis on who the *Cx3cr1* expressing cells are during acute infection **supplemental figure 5h-j & supplemental figure 5m** we are uncertain which cell population or which tissue to target for an adoptive transfer. Additionally, due to the homeostatic niche of macrophages (still a putatively important population), adoptive transfer of macrophages/monocytes is generally not successful unless mice undergo lethal irradiation *(Hashimoto, D., et al., 2013 Cell press).* After successful repopulation from monocyte derived cells, the monocyte derived macrophages are distinct and in some scenarios respond and generate inflammation differently from the original macrophages (*Rua et al., 2019. Nat Immunol*) (*Louwe et al., 2021. Nat Commun*). It is possible that a monocyte derived macrophage may have completely different inflammasome mediated response from the original embryonically-derived tissue macrophage. Because of the additional unknowns/variables involved in completing this experiment, and our revised conclusions, we do not think this experiment is likely to be successful.

3. To know during in vivo T. gondii infection if direct invasion of the parasites is required or host sensing/ parasite attachment/protein secretion is sufficient for Caspase 1 activation to release IL-18 the authors should perform a similar experiment as Fig 1f using heat killed parasites.

These are interesting studies, but we believe they are outside the scope of the current manuscript. Additionally, as we are uncertain that heat killed parasite or soluble *T. gondii* antigen (STAG) would be a sufficient tool. STAG induces a short-lived, less than 24hr, intense IL-12 mediated IFN-γ response and leads to dysfunctional DCs thus ultimately not mimicking the dynamics of *T. gondii* infection *(Sousa et al., 1997. JEM) (Sousa et al., 1999. Cell press).* We agree with the reviewer that understanding how *T. gondii* activates the inflammasome in vivo is an important area of study and to thoroughly address this question it would require extensive experimentation and a separate manuscript.

4. Authors should include a gating strategy which was used to define different immune cells populations (T cells, Mono, Neutrophils, NK/ ILC1/ NK T cells) in the supplementary figure for better understanding of the reader.

Thank you for this helpful suggestion. We have included a full gating strategy in **supplemental figure 1f through supplemental figure 1u.**

Clarifications not requiring data

5. it seems possible that the microbiota has a role in the secretion of IL-18 by caspase 1. This could be addressed by examining the parasite burden in wild type C57Bl6 and caspase 1 ko mice with and without antibiotic s. Alternatively, the authors should acknowledge this limitation and suggest follow up studies in the Discussion.

We have included a statement in the discussion addressing how the microbiota might impact the activation of the inflammasome and thus impact effects seen from *Casp1* KO mice on **line 385-387**.

6. Please explain in the discussion why CD4+ T cell IFN-γ production is significant in Casp1 KO mice in spleen but not in the peritoneum (Fig 1 and Suppl Fig S1).

We have included a sentence in the discussion on **line 360-363** addressing our hypothesis for why we did not observe a defect in $CD4^+$ T cell IFN-γ production in the peritoneum.

Reviewer #2:

1. A detailed FACS analyses of lymphoid and myeloid populations in peritoneum and spleen of infected the mice is required.

We have included data on the activation state**(supplemental figure 2a,f and 4e,i),** T-bet expression of CD4 and CD8 T cells in both tissues **(supplemental figure 2c,i and 4g,l),** monocyte and neutrophil frequency in the peritoneum **(supplemental figure 2l-m and 3g-h),** iNOS frequency in peritoneum **supplemental figure 2n, 3i, and 4m** and iNOS MFI in peritoneum **supplemental figure 2o, 3j, 4n**.

2. Characterize the population of Caspase-1 expressing Cx3cr1+ cells.

We have generated a **new supplemental figure 5** that characterizes *Cx3cr1*$^{+}$ cells throughout the acute infection in **supplemental figure 5a-j.**

3. Demonstrate that IL-18 expression is upstream (of IFN-g) and not down-stream from IFNgR-gamma signaling.

We acknowledge that IFN-gamma production may affect IL-18 expression, but ultimately, we observe defects in IL-18 release and CD4 T cell IFN-gamma production. This specific request would require interfering with IFN-gamma signaling, which would complicate the interpretation of results as uncontrolled parasite replication would also occur. In the interpretation of our results, we are agnostic to whether IL-18 expression is upstream or downstream of IFN-γ signaling.

Reviewer #3:

1. The differences in IFN-g levels were reported to be selectively significant for CD4 T cells (Figure 1F), but not for other cell types (supplementary data). However, a closer inspection of the data shows that there may also be changes (increases/decreases) in CD8 T cells, NK and ILCs which may not have reach statistical significance. It would be better to display the p-values, rather than denoting these as non-significant. The overall decreases in IFN-g levels is about 20-30%, but the levels produced in caspase-deficient mice are still very high and apparently sufficient to control acute infection and allow for establishment and maintenance of chronic infection. Thus, it is not clear to what extent the additional production of IFN-g by CD4 T cells is beneficial overall to host resistance/disease tolerance. The flip side of the argument is that increased IFN-g production by CD4 T cells induced by caspase-1/IL-18 likely increases acute immunopathology and could further contribute to inflammation of the CNS in the chronic phase. Did the authors track the weight changes during infection and did the authors examine whether caspase-1/IL-18 deficiency ameliorated chronic symptoms?

We have included p-values for all data in the manuscript.

Thank you for the thoughtful alternative hypothesis, we agree that excessive IL-18 and IFN-γ production can lead to immunopathology and this has been previously reported *(Clark et al., 2024. Cell Rep and Vossenkämper et al., 2004. Eur J Immunol).* The lack

of death seen in *Casp1* KO mice is interesting and may be due in part to preventing cachexia induced by IL-1R signaling (Melchor et al., *2020. J Immunol*), which is downstream of caspase-1 activity [1]. Additionally, have included weight changes for *Casp1* KO mice and WT mice throughout infection in **supplemental figure 1e** and observe that Casp1 KO mice are protected from infection induced weight loss. We have revised the text on **line 132-134** to reflect this finding.

2. The authors utilized the Cx3cr1 cre system to delete Caspase 1 and call them "macrophage Casp1 knockout" mice. However, they do not clearly delineate the cell types affected by this maneuver nor discuss how to interpret the results obtained. Which specific myeloid/lymphoid cell derived lineages are affected here? cDC-1, CCR2+ monocytes and their derivatives and tissue macrophages?

We have included a **new supplemental figure 5** where we track the *Cx3cr1*$^{+}$ populations during acute *T. gondii* infection. Results generated suggest that throughout the acute phase many different cell types express *Cx3cr1*$^{+}$ population at a given time/tissue. Given this new data we have changed our nomenclature throughout the text to refer to Cx3cr1-cre caspase-1$^{fl/fl}$ mice as *Cx3cr1*$^{+}$ *Casp1* KO mice.

Part III – Minor Issues: Editorial and Data Presentation Modifications
Please use this section for editorial suggestions as well as relatively minor modifications of existing data that would enhance clarity.

Reviewer #1: 1. Title- The main finding of the study is the role of Caspase in Cx3cr1 expressing macrophages during infection so, it would be better to have cx3cr1-expressing macrophages in the title. Also change T. gondii to Toxoplasma gondii.

We were unable to confirm that the pertinent cell type is a macrophage, although it seems likely, so we have refrained from describing the effects we see as being dependent on a macrophage caspase-1 and instead refer to it as a Cx3cr1+ cells *Casp1*.

We have changed the title to from *T. gondii* to Toxoplasma gondii.

2. Line 146- Correct ICL1 to ILC1.

Done, thanks!

3. Line 164- Add a short background to explain the rationale of the experiment. "A recent study from Clark et. al., 2023, Cell Report describes that IL-18, and its regulation impacts the ability of CD4+ T cells to make IFN-γ".

We agree. We have done so, can be seen on **line 175-176**.

4. Line 234- Change S3b-S3d Fig to S3d-S3f.

Figures have been corrected!

5. *Line 270- A study by Sateriale et al, 2020, PNAS has shown that caspase-1 is sufficient to provide innate resistance to cryptosporidiosis when compared to Casp1/11 KO mice. This reference can be added in the discussion to support your study.*

We have added this to our discussion **line 303-305** to strengthen the support for our study, thank you!

6. In the Figure 3i- change heading from Ly6G+ to Neutrophils.

Done.

7. Line 704- In the figure legend change (j) to (g).

Figure legends have been corrected.

Reviewer #2: Figure formatting should be improved (e.g. Figure 2: Peritoneum day 8 post ?).
Using scientific notation would also be helpful when showing absolute cell numbers.

Figure formatting has been improved, thanks!

Reviewer #3: (No Response)
